# Phytochemical Composition and Antimicrobial Properties of New *Lavandula angustifolia* Ecotypes

**DOI:** 10.3390/molecules29081740

**Published:** 2024-04-11

**Authors:** Izabela Betlej, Bogusław Andres, Tomasz Cebulak, Ireneusz Kapusta, Maciej Balawejder, Natalia Żurek, Sławomir Jaworski, Agata Lange, Marta Kutwin, Elżbieta Pisulewska, Agnieszka Kidacka, Barbara Krochmal-Marczak, Piotr Boruszewski, Piotr Borysiuk

**Affiliations:** 1 Institute of Wood Sciences and Furniture, Warsaw University of Life Sciences—SGGW, 159 Nowoursynowska St., 02-776 Warsaw, Poland; boguslaw_andres@sggw.edu.pl (B.A.); piotr_boruszewski@sggw.edu.pl (P.B.); 2 Department of Food Technology and Human Nutrition, Institute of Food Technology and Nutrition, College of Natural Sciences, University of Rzeszów, 4 Zelwerowicza St., 35-601 Rzeszów, Poland; tcebulak@ur.edu.pl (T.C.); ikapusta@ur.edu.pl (I.K.); nzurek@ur.edu.pl (N.Ż.); 3 Department of Chemistry and Food Toxicology, University of Rzeszów, 1a Ćwiklińskiej St., 35-601 Rzeszów, Poland; mbalawejder@ur.edu.pl; 4 Department of Nanobiotechnology, Institute of Biology, Warsaw University of Life Sciences, 8 Ciszewskiego St., 02-786 Warsaw, Poland; slawomir_jaworski@sggw.edu.pl (S.J.); agata_lange@sggw.edu.pl (A.L.); marta_prasek@sggw.edu.pl (M.K.); 5 Department of Plant Production and Food Safety, Carpathian State College in Krosno, 38-400 Krosno, Poland; elzbieta.pisulewska@gmail.com (E.P.); barbara.marczak@pans.krosno.pl (B.K.-M.); 6 Breeding Department, Małopolska Plant Breeding Company sp. z o. o., 4 Zbożowa St., 30-002 Kraków, Poland; akidacka@mhr.com.pl

**Keywords:** *Lavandula angustifolia*, bioactive compounds, ethanolic extracts, antimicrobial activity, GC-MS analysis, UPLC chromatography

## Abstract

The purpose of this study was to characterize ethanol extracts from leaves and flowers of two ecotypes (PL—intended for industrial plantations and KC—intended for cut flowers) of *Lavandula angustifolia* Mill. The plant was cultivated in 2019 in southern Poland as part of a long-term research plan to develop new varieties resistant to difficult environmental conditions. The collected leaves and flowers were used to prepare ethanol extracts, which were then analyzed in terms of phytochemical composition and antioxidant, bactericidal, and fungicidal properties. Using UPLC techniques, 22 compounds belonging to phenolic acids and flavonoids were identified. UPLC test results indicated that ethanol extracts from leaves and flowers differ in phytochemical composition. Lower amounts of phenolic acids and flavonoids were identified in leaf extracts than in flower extracts. The predominant substances in the flower extracts were rosmarinic acid (829.68–1229.33 µg/g), ferulic acid glucoside III (810.97–980.55 µg/g), and ferulic acid glucoside II (789.30–885.06 µg/g). Ferulic acid glucoside II (3981.95–6561.19 µg/g), ferulic acid glucoside I (2349.46–5503.81 µg/g), and ferulic acid glucoside III (1303.84–2774.17 µg/g) contained the highest amounts in the ethanol extracts of the leaves. The following substances were present in the extracts in trace amounts or at low levels: apigenin, kaempferol, and caftaric acid. Leaf extracts of the PL ecotype quantitatively (µg/g) contained more phytochemicals than leaf extracts of the KC ecotype. The results obtained in this study indicate that antioxidant activity depends on the ecotype. Extracts from the PL ecotype have a better ability to eliminate free radicals than extracts from the KC ecotype. At the same time, it was found that the antioxidant activity (total phenolic content, ABTS^•+^, DPPH^•^, and FRAP) of PL ecotype leaf extracts was higher (24.49, 177.75, 164.88, and 89.10 μmol (TE)/g) than that determined in flower extracts (15.84, 125.05, 82.35, and 54.64 μmol (TE)/g). The test results confirmed that leaf and flower extracts, even at low concentrations (0.313–0.63%), significantly inhibit the growth of selected Gram-negative and Gram-positive bacteria and Candida yeasts. Inhibition of mold growth was observed at a dose extract of at least 1 mL/100 mL.

## 1. Introduction

The genus Lavandula includes about 50 species cultivated around the world as an ornamental and medicinal plant [1]. This plant comes from the Mediterranean area, the Middle East, North Africa, and the Republic of Cape Verde. It is also successfully cultivated in Asia, Australia, and the Americas. In Europe, the main production of lavender is concentrated in the regions of France, Bulgaria, Spain, and Ukraine. French lavender, and in fact, the raw material obtained from it, is an important item in the economic balance of this country [2].

The first plantations of this species in the climatic and soil conditions of southern Poland were already carried out in the 1920s [3,4]. The currently observed changes in weather conditions, especially the occurrence of mild winters, enable the cultivation of lavender with a longer flowering period, which may last until the end of October. One of the most frequently chosen lavender species for cultivation in Poland is *Lavandula angustifolia* Mill. Its numerous foreign varieties are valued, such as Siesta, Beate, Essence, Blue Scent, and Hidcote Blue Strain [5]. Most of these varieties show relatively high resistance to low temperatures, so they can be successfully cultivated in Poland, unlike *Lavandula stoechas* L., which does not tolerate negative temperatures and freezes in winter in field cultivation [6]. Despite numerous plantations in various regions of Poland, there is still no Polish breeding variety.

The most valuable raw material, *Lavandula angustifolia* Mill. is flowers containing essential oil that is valuable for the cosmetic and pharmaceutical industry. Shi [7] indicated that essential oil contains over 100 individual components with aromatic and biological effects. The dominant substances in the oil are linalool, linalyl acetate, cineole, β-ocimene, β-lavender acetate, lavender alcohol, terpene-4-alcohol, and camphor [7,8]. At the same time, significant variability in the composition of the oil was demonstrated, both among different varieties of lavender and the method of obtaining it [9]. Adaszyńska et al. [10] compared the chemical composition of the essential oil of 5 varieties is *L. angustifolia* Mill. originating from controlled crops, and they observed significant differences in the quantitative composition of the main components of the oil and chemical substances occurring in small concentrations. Moreover, the method of extraction and the selection of raw materials (leaves, flowers, and inflorescence stalks) allows for the attainment of oils of different quality [11]. For example, in the research conducted by Dvorackova et al. [12] and Bajkacz et al. [13], it was shown that the leaves of *L. angustifolia* Mill. are richer in hydroxycinnamic acids than the flowers of this plant. In turn, Dobros et al. [14] showed that the extraction method plays an important role in the final content of bioactive substances and antioxidant properties.

In addition to terpenoid components, lavender contains numerous polyphenols, coumarins, sterols, and tannins [15,16,17]. Undoubtedly, important chemical compounds present in lavender flowers are phenolic compounds, which are believed to have an antioxidant role. Yadikar et al. [18] proved the strong antioxidant effect of polyphenolic compounds such as lavandunat, lavandufurandiol, lavanduflu-oren, lavandupyrones, lavandudiphenyls, 4-(1-hydroxy-1-methylethyl) benzoic acid, methyl 3-(3,4-dihydroxyphenyl) propanoate, 3,4,α-trihydroxyl-ethyl phenylpropionate, rosmarinic acid, and isosalvianolic acid, isolated from the flowers of *L. angustifolia* Mill. in the steam distillation process.

The composition and proportions of bioactive ingredients in extracts obtained from lavender raw materials determine its biological functions and potential applications. Due to its proven antibacterial [19], antifungal [20], antioxidant [21], and anti-inflammatory [22] properties, this plant is very popular among both breeders and potential recipients of the raw material. Numerous scientific publications devoted to the chemical analysis of the raw material also demonstrate great interest [23,24]. Lavender is therefore becoming more and more popular in cultivation, as well as in regions where it was rarely found before. New growing conditions also make it possible to obtain new varieties that will give high yields in climatic and soil conditions previously considered unfavorable for the plant’s vegetation. At the same time, the chemical characteristics of the raw material and its pharmacopoeial significance are important, as they will show that the raw material obtained from a cold temperate climate is characterized by broad biological activity, which may constitute an alternative to the raw material obtained from regions typical for its cultivation.

The aim of the study was to assess the phytochemical composition and antioxidant and antimicrobial activity of ethanol extracts from flowers and leaves of two ecotypes of *Lavandula angustifolia* Mill. (marked with PL and KC symbols) cultivated in southern Poland since 2019. The presented lavender ecotypes were selected for the first time and adapted to the soil and climatic conditions of Poland. So far, no one has tested the antioxidant, antifungal, and antibacterial properties of the presented ecotypes. The cultivation of narrow-leaved lavender in the form of ecotypes of a medium-high plant and a bouquet plant is intended in the future to lead to the separation of a new variety, adapted to cultivation in regions dominated by low temperatures in winter. However, for lavender to be a good raw material that meets quality standards for the cosmetics and pharmaceutical industry, the composition of biologically active substances must be thoroughly characterized, and its properties have to be examined.

## 2. Results

### 2.1. Phytochemical Compounds

UPLC analysis showed that eight phenolic acids and two flavonoid compounds were identified in leaf extracts (PL1 and KC1). As many as 14 phenolic acids and 6 flavonoid compounds have been identified in flower extracts (PL2 and KC2). Although leaf extracts are less diverse in phenolic compounds, it should be noted that, quantitatively, these compounds significantly exceed the content of phenolic compounds identified in flower extracts. The content of ferulic acid glucose II in the extracts from PL1 leaves was almost ten times higher than in the extracts from PL2 flowers. Similarly, the content of substances such as caftaric acid, isorhamnetin-3-*O*-rhamnoside, and ferulic acid was much higher in leaf extracts than in lavender flower extracts (Table 1). Substances such as glucoside I, caffeic acid, chicoric acid, kaempferol, salvinic acid B, apigenin glucoside C, and apigenin glucoside C were not determined in trace amounts in leaf extracts. These substances were identified in flower extracts. Ferulic acid glucoside I and isorhamnetin 3-*O*-rutinoside were not determined in trace amounts in flower extracts. These substances were identified in leaf extracts in amounts of 243.81 µg/g (KC1) and 621.76 µg/g (PL1). The tests performed showed that extracts from ecotype PL contained higher amounts of phenolic compounds than extracts from ecotype KC (Table 1).

GC-MS analysis showed qualitative and quantitative differences in the composition of volatile substances between ecotypes. Decane, lavandulyl acetate, and β-farnesene were identified only in extracts of the KC ecotype (Table 2), while myrcene was only identified in the PL ecotype (Table 2). Linalool and linalyl acetate, the main components of the essential oil, were identified only in flower extracts, with the KC ecotype containing much larger amounts of the indicated substances than the PL ecotype. The number of volatile substances in flower extracts was almost twice as high as in leaf extracts. 

### 2.2. Antioxidant Assay

The analysis of the antioxidant properties of lavender leaf and flower extracts was carried out based on the ability of the extracts to reduce radicals (ABTS^•+^ and DPPH^•^) and iron (FRAP). The total content of phenolic compounds (TPC) was also measured. Based on the analyses performed, it was found that the extract from the PL ecotype leaves has a strong antioxidant effect (Table 3). There were no statistically significant differences in antioxidant properties between flower extracts. The leaf extract of the KC ecotype showed the weakest ability to reduce the DPPH^•^ radical, iron and it contained a relatively small amount of TPC.

In all cases (total phenolic content, ABTS^•+^, DPPH^•^, and FRAP), the influence of the analyzed factors (ecotype and L/F extract) and the interactions between them showed a statistically significant effect (*p* < 0.05). Only in the case of ABTS^•+^ was the effect of the interaction between ecotype and L/F extract statistically insignificant. By analyzing the influence of individual factors, it should be concluded that in relation to total phenolic content, DPPH^•^, and FRAP, the greatest influence is exerted by the ecotype (38.0–47.7%) and the interaction between the ecotype and the type of extract (46.3–57.0%). The type of extract is important (61.6%) only in relation to ABTS^•+^; in other cases, the influence of this factor is much lower (0.4–14.5%). It should also be emphasized that in all cases, there was only a slight impact of factors not taken into account in these studies (error 0.1–0.5%) (Table 4).

### 2.3. Measurement of the Growth of Mold Fungi and Bacterial Viability

Table 5 and Table 6 show the effect of extracts on the growth of mold fungi. Studies have shown that leaf extracts at a concentration of 1 mL/100 mL completely inhibit the growth of *Trichoderma viride* and *Chaetomium globosum*. Lavender flower extracts had a weaker fungicidal effect, especially against *T. viride*.

**Figure 1:**
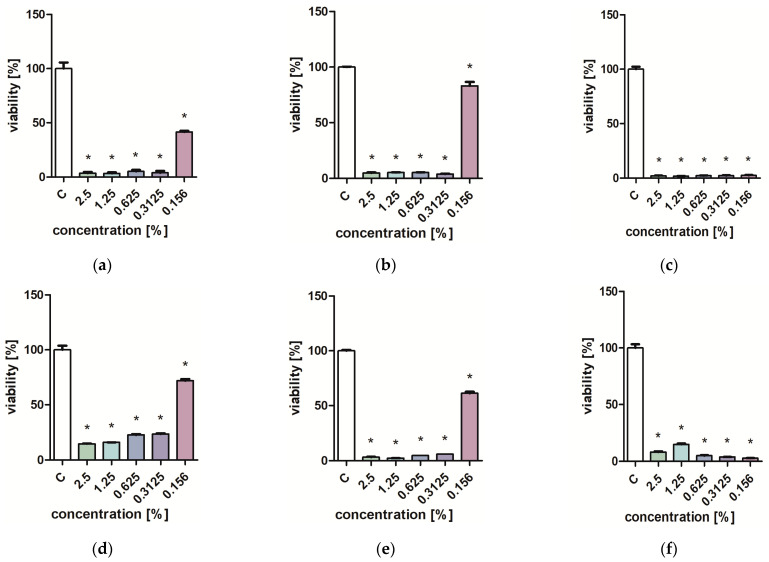
Effect of PL1 extracts on the viability (**b**,**e**) *P. aeruginosa*; (**c**,**f**) *S. aureus*. PrestoBlue test (**a**–**c**) and XTT test (**d**–**f**). Results are presented as mean values ± standard deviation. Asterisks indicate a statistical significance level at *p*-value ≤ 0.05.

**Figure 2:**
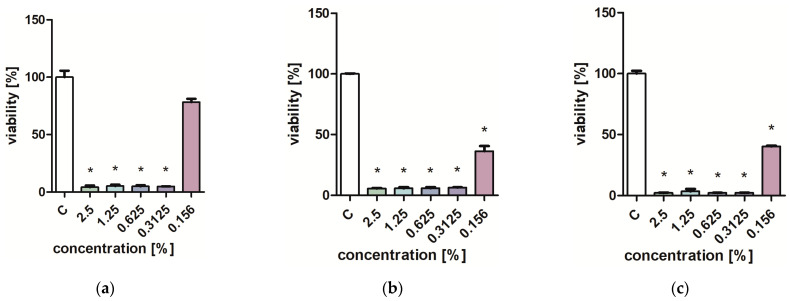

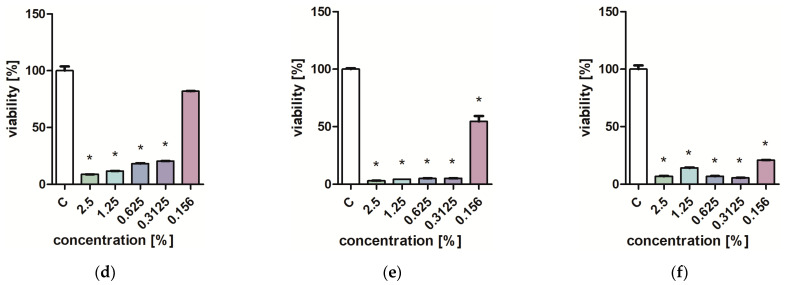
Effect of KC1 extracts on the viability (**a**,**d**) *C. albicans*; (**b**,**e**) *P. aeruginosa*; (**c**,**f**) *S. aureus*. PrestoBlue test (**a**–**c**) and XTT test (**d**–**f**). Results are presented as mean values ± standard deviation. Asterisks indicate a statistical significance level at *p*-value ≤ 0.05.

**Figure 3:**
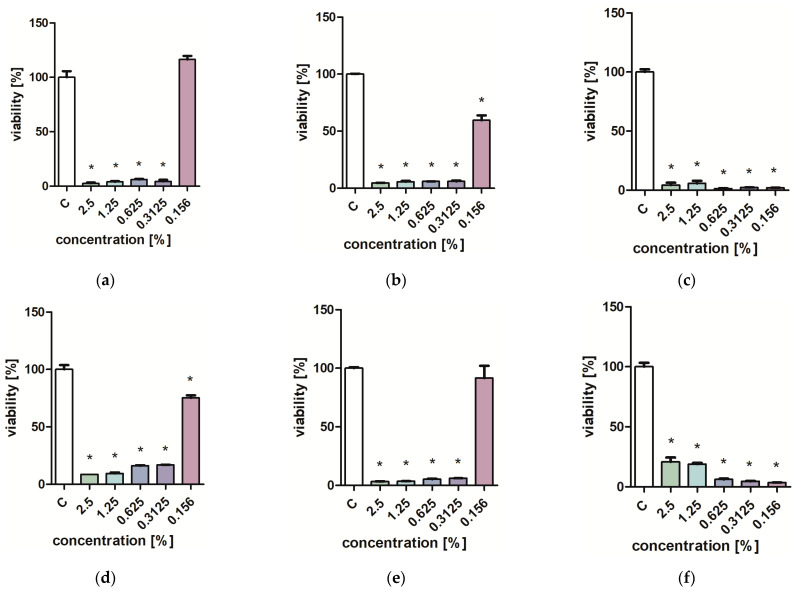
Effect of PL2 extracts on the viability (**a**,**d**) *C. albicans*; (**b**,**e**) *P. aeruginosa*; (**c**,**f**) *S. aureus*. PrestoBlue test (**a**–**c**) and XTT test (**d**–**f**). Results are presented as mean values ± standard deviation. Asterisks indicate a statistical significance level at *p*-value ≤ 0.05.

**Figure 4:**
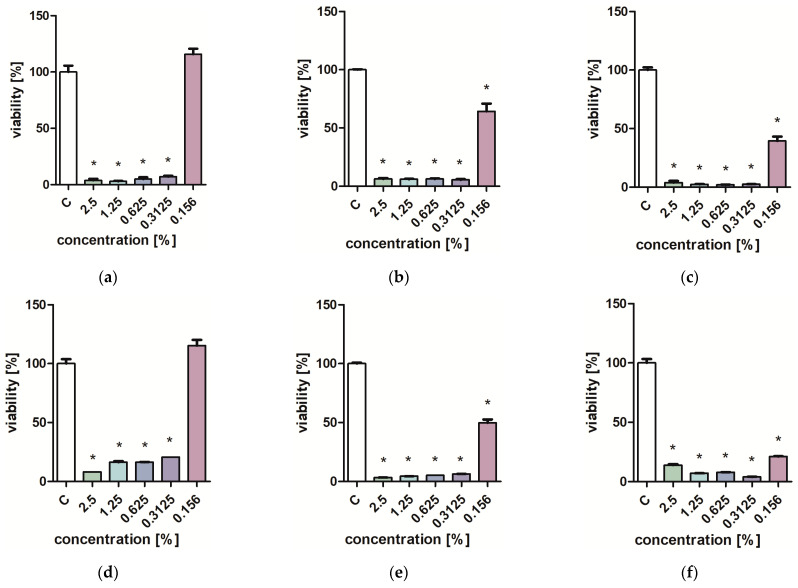
Effect of KC2 extracts on the viability (**a**,**d**) *C. albicans*; (**b**,**e**) *P. aeruginosa*; (**c**,**f**) *S. aureus*. PrestoBlue test (**a**–**c**) and XTT test (**d**–**f**). Results are presented as mean values ± standard deviation. Asterisks indicate a statistical significance level at *p*-value ≤ 0.05.

Studies on the influence of extracts on the viability of *Pseudomonas aeruginosa, Staphylococcus aureus,* and *Candida albicans* yeast cells showed that low concentrations of extracts (0.313–0.625%) inhibit the growth of microorganisms (Figure 1, Figure 2, Figure 3 and Figure 4). Particularly strong antibacterial activities of leaf extracts (PL1 and KC1) were demonstrated in the PrestoBlue test (Figure 1 and Figure 2). A percentage of 0.156% leaf extract of the PL ecotype turned out to be a strong inhibitor of the growth of *S. aureus* (Figure 1). Flower extracts (PL2 and KC2) also showed a strong effect on microbial viability (Figure 3 and Figure 4). Ethanol used in analogous concentrations did not inhibit the tested mold fungi.

### 2.4. Graphical Interpretation of Results

**Figure 5:**
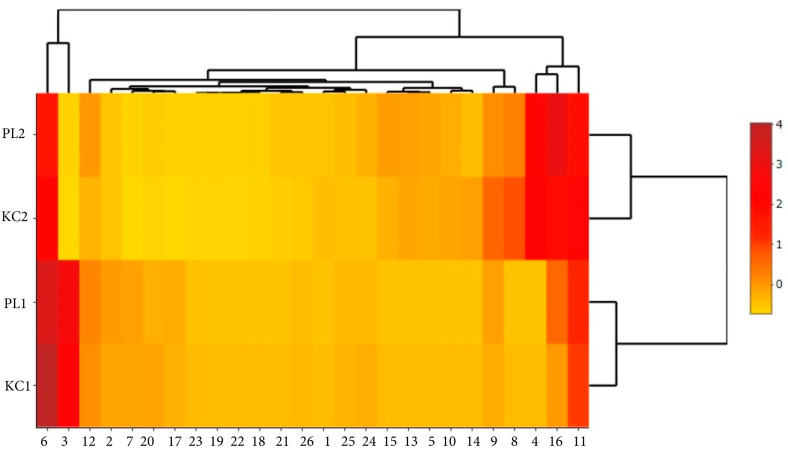
Imaging of the diversity of the content of polyphenolic compounds and antioxidant activities in PL and KC lavender ecotypes using a heat map. (1)—Syringic acid glucoside, (2)—Caftaric acid, (3)—Ferulic acid glucoside I, (4)—Coumaric acid glucoside I, (5)—Caffeic acid, (6)—Ferulic acid glucoside II, (7)—Isorhamnetin 3-*O*-rutinoside, (8)—Apigenin 4′-*O*-glucoside-7-*O*-glucuronide, (9)—Coumaric acid glucoside II, (10)—Chicoric acid, (11)—Ferulic acid glucoside III, (12)—Isorhamnetin 3-O-rhamnoside, (13)—(+)Catechin-rhamnoside-pentoside, (14)—Salvinic acid B, (15)—Apigenin C-glucoside, (16)—Rosmarinic acid, (17)—Ferulic acid, (18)—Unidentified caffeic acid derivative, (19)—Kaempferol, (20)—Undefined caffeic acid derivative, (21)—Undefined caffeic acid derivative, (22)—Apigenin, (23)—TPC, (24)—ABTS, (25)—DPPH, (26)—FRAP.

**Figure 6:**
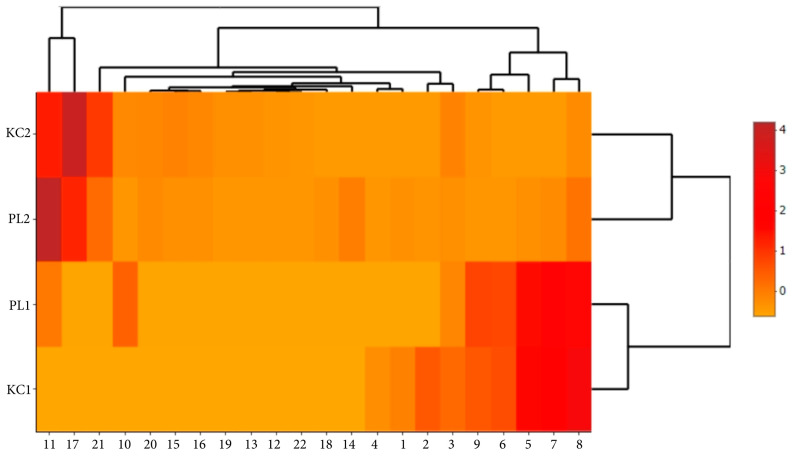
Imaging of the differences in the content of volatile compounds in PL and KC lavender ecotypes using a heat map. (1)—α-Pinene, (2)—Camphene, (3)—β-Pinene, (4)—Decane, (5)—3-Carene, (6)—p-Cymene (cymene isomers mix), (7)—m-Cymene (cymene isomers mix), (8)—Limonene, (9)—Eucalyptol, (10)—γ-cadinene, (11)—Ocimene isomers mix, (12)—3-octanone, (13)—o-Cymene, (14)—Myrcene, (15)—Linalool, (16)—1-Octen-3-yl acetate, (17)—Linalyl acetate, (18)—2,6-Dimethyl-2,4,6-octatriene, (19)—Lavandulyl acetate, (20)—α-santalene, (21)—B-Caryophyllene, (22)—B-Farnesene.

**Figure 7:**
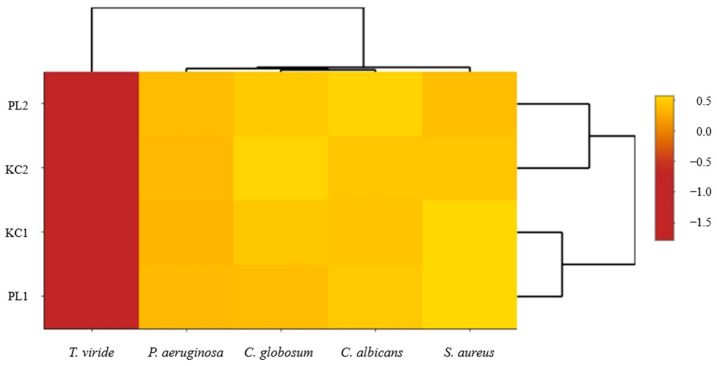
Heat map imaging of antimicrobial properties, expressed as colony reduction efficiency of extracts belonging to PL and KC ecotypes.

Mapping the data obtained from the tested material using scaled heat maps shows a clear differentiation of qualitative and quantitative characteristics of two lavender ecotypes KC2, PL2 to KC1, and PL1. This differentiation is clearly visible in the case of polyphenolic compounds (Figure 5), volatile compounds (Figure 6), and antimicrobial activity (Figure 7).

The use of a heat map with a scaling function enabled a more accurate presentation of the relationships between the analyzed factors within ecotypes and specific extracts. It is clearly visible that leaf extracts (KC1 and PL1) are characterized by greater activity of ferulic acid glucoside I, caftaric acid, and isorhamnetin 3-*O*-rutinoside (Figure 5). Flower extracts (KC2 and PL2) are richer in volatile substances such as ocimene isomers mix, linalyl acetate, and β-caryophyllene. In turn, lavender leaf extracts have a greater ability to eliminate free radicals (Figure 6). The heat map analysis showed that the microbiological activity within ecotypes and extracts is similar (Figure 7). The tested ecotypes showed very strong moldicidal activity against *T. viride*. Lavender leaf extracts had a weaker effect on the viability of *S. aureus* than lavender flower extracts.

## 3. Discussion

Lavender is a plant of high pharmacopoeial value. Thanks to numerous bioactive ingredients, it is an important raw material for the pharmaceutical, cosmetics, and food industries. The quality of the raw material depends largely on the species but also on the climatic and soil conditions in which it is grown. Due to the observed changes in the environment related to climate warming, it has become possible to grow and process *Lavandula angustifolia* Mill. in regions that were not favorable for its cultivation until recently. The economic value of the raw material depends on its quality related to the expected properties. The quality of the lavender raw material, understood as the quality of the crop, giving a high content of essential oil and bioactive ingredients with biological properties, is the subject of numerous analyses [25,26,27]. Crişan et al. [28] indicated that the phytochemical profile determines the functional potential of lavender. Da Porto and Decorti [29] showed that in extracts from *L. angustifolia* Mill. originating from cultivation in Italian regions, the dominant ingredients are linalyl acetate, linalool, and a low percentage of camphor. The characteristic ingredients of Iranian lavender are linalool, linalyl acetate, lavandulyl acetate, α-terpineol, and geranyl acetate [30]. This study showed that *L. angustifolia* Mill. grown in southern Poland is dominated by 1-octen-3-yl acetate, ocimene isomers mix, γ-cadinene, and limonene. In addition to the main components of the oil, lavender extracts contain antioxidant compounds. The conducted analyses identified 22 compounds belonging to phenolic acids and flavonoids. In research conducted by Tundis et al. [31], the dominant polyphenolic compounds in water–ethanol extracts from *L. angustifolia* Mill. were rosmarinic acid, ferulic acid glucoside, morin, and caffeic acid. The results of our research confirm the reports of other researchers. Rosmarinic acid was the dominant component in leaf and flower extracts. Apigenin, kaempferol, and caftaric acid were present in the extracts in trace amounts or at low levels. In studies by other authors, substances found in trace amounts were chlorogenic acid and ferulic acid [32].

In our own research, extracts from *L. angustifolia* Mill. flowers were characterized by a greater variety of bioactive ingredients than leaf extracts. Differences in qualitative composition were also observed between ecotypes. Bioactive compounds, especially polyphenols, determine the antioxidant potential [33]. Scientific research indicates that extracts obtained from raw lavender materials can prevent the damage of oxidative cells [34]. The presented research results proved that the antioxidant activity depends on the ecotype. The PL ecotype has a better ability to eliminate free radicals than the KC ecotype. Additionally, it can be concluded that the obtained results of antioxidant properties are at a satisfactory level. The best antioxidant properties were demonstrated for ethanol extracts from leaves and flowers of the PL ecotype. The antioxidant potential of extracts from the PL ecotype was in the range of 125.05–177.75 μmol(TE)/g (ABTS^•+^), 82.35–164.88 mol(TE)/g (DPPH), and 54.64–89.10 μmol(TE)/g (FRAP). The scientific literature contains extensive data on the antioxidant properties of various *L. angustifolia* extracts. Caser et al. [1] showed that the antioxidant potential of fresh and dried flowers of *L. angustifolia* Mill. assessed in the ABTS method ranges from 17.89 to 44.81 μmol TE/g and in the DPPH method from 16.06 to 47.36 μmol TE/g. In turn, Robu et al. [35] showed that the highest antioxidant value of water–ethanol extracts does not exceed 110.36 µg/mL.

The antimicrobial properties of various lavender extracts have been documented in numerous scientific studies [36,37,38]. de Rapper et al. [39] demonstrated the synergistic effect of combinations of essential oil with selected antibiotics against representative bacterial cells (*Staphylococcus aureus* and *Pseudomonas aeruginosa*) and yeast (*Candida albicans*). A comprehensive review of the properties of *L. angustifolia* Mill. extracts. conducted by Salehi et al. [40] indicates the crucial importance of lavender as an antibacterial, antifungal, and antiseptic raw material. These tests also confirmed high biocidal properties of low concentrations of extracts against selected Gram-negative and Gram-positive bacteria, fungi of the *Candida* genus, and mold fungi. 

The conducted research demonstrated the relationship between ecotypes and plant parts and the identified phytochemical composition and biological properties. Therefore, it can be assumed that there is a relationship between the phytochemical composition of specific extracts and ecotypes and their biological properties. The results presented in the form of heat maps may suggest that certain groups of substances contained in ecotypes and in specific parts of plants may have an effect on the viability of bacteria and mold fungi. Substances such as ferulic acid glucoside I, ferulic acid glucoside II, ferulic acid glucoside III, or rosmarinic may have antimicrobial effects in flower and leaf extracts. These assumptions seem to be correct from the point of view of the results of other researchers, who point to the enormous antimicrobial potential of these substances [41]. Similar conclusions can be drawn by analyzing the impact of ecotypes and the type of extract on antioxidant activities. Heat map analysis indicates that the antioxidant properties of the PL ecotype may be related to a different phytochemical composition than the antioxidant properties of the KC ecotype.

Lavender ecotypes grown in the Polish climate are plants with good antioxidant and biological potential, which may translate into their high use in various industries where such properties are desired. The presented work showed that extracts prepared from lavenders are not rich in linalool, which dominates in lavenders from Mediterranean countries [42]. Research presented by Despinasse et al. [43] indicates the division of Mediterranean lavenders into three chemotypes, differing in the dominance of phytochemical components. The tested PL ecotype was dominated by volatile components such as m-cymene, limonene, and ocimene isomers mix. In turn, the KC ecotype contained the highest percentage of volatile components, such as linalyl acetate and limonene. Therefore, it can be concluded that the PL ecotype differs significantly from the most frequently cultivated French varieties, which are dominated by linalool and linalyl acetate. In turn, the dominance of limonene in the KC ecotype suggests that its origin in French lavenders can be ruled out. The ISO3515:2004 standard characterizes the composition of French *L. angustifolia*, in which the content of limonene in the oil fraction does not exceed 0.5% [44]. It should also be mentioned that French lavenders are very difficult to grow in the difficult climate of winter seasons in Poland; therefore, breeding varieties for which the climatic conditions prevailing in southern Poland allow for growth and are additionally characterized by a rich phytochemical composition is extremely important. From an agronomic point of view, it is very important. 

Comparing the biological properties of new lavender ecotypes with varieties grown in the Mediterranean region, it should be concluded that they are comparable [45]. Similarly to lavenders from the Mediterranean regions, the oxygenated monoterpenes and phenolic compounds contained in them are responsible for the antimicrobial properties against pathogens such as *P. aeruginosa* and *S. aureus* [46].

The quantitative and qualitative composition of phytochemical components of the tested lavender ecotypes provides extremely valuable knowledge about the quality of the raw material. It is believed that the Polish climate, especially the temperature and humidity conditions prevailing in the winter and spring, are a factor influencing the high content of selected volatile fractions, but for these data to be confirmed as reliable, many years of agronomic research are required. It should be additionally mentioned that the phytochemical composition of two lavender ecotypes analyzed in the presented work may also have a huge impact on its sensory properties, which should be assessed in subsequent research tasks. 

## 4. Materials and Methods

### 4.1. Characteristics of the Research Material

The research material included two ecotypes of lavender (*Lavandula angustifolia* Mill.): a medium-height plant form intended for cultivation as a flower on production plantations for the pharmaceutical, cosmetics, or perfume industry (PL) and a bouquet form, developing long-stemmed inflorescences, elongated racemes, and less frequently set pseudo circles (KC). The field experiment was carried out in Polanowice (50°19′ N 20°07′ E) at the Plant Breeding Station belonging to the Małopolska Plant Breeding Station. The research was carried out on chernozem soil (bonite class 1) with a limestone substrate (pH 6.8). Mineral fertilization was applied in spring before planting. Seedlings obtained generatively (from seeds) were planted on 19 June 2019 in 9-point strips with a spacing of 0.5 × 0.5 m. A total of 91 rows. Three samples of flowers and leaves, each weighing 100 g, were collected from three-year-old plants (7 July 2021) of each ecotype.

Weather conditions varied during the growing season in 2019–2021. The years 2019 and 2020 were similar in terms of rainfall (646.3 and 652.75 mm, respectively), and in 2021, 200 mm more was recorded (845 mm). Their distribution was also different. In 2019, most rain occurred in May and August; in 2020, in May and June; and in 2021, in July and August. In turn, the warmest year was 2019, and the highest average temperatures occurred in June, July, and August.

The leaves and flowers were divided into portions of 10 g each and then placed in vessels to which 200 cm^3^ of 60% ethanol was added. The whole thing was left to macerate for 72 h. After this time, the extracts were separated from the leaves and flowers and purified using syringe filters with a pore diameter of 0.22 µm. Leaf extracts were given the symbols PL1 and KC1, and flower extracts PL2 and KC2. Extracts for testing were stored at 2–3 °C.

### 4.2. Phytochemicals Analysis

#### 4.2.1. Phenolic Compound Identification

The determination of polyphenolic compounds was carried out using the ultra-performance liquid chromatography (UPLC-PDA-MS/MS) Waters ACQUITY system (Waters, Milford, MA, USA), according to the previous article [47]. The UPLC system (UPLC-PDA-MS/MS) was equipped with a binary pump manager, column manager, sample manager, photodiode array (PDA) detector, and tandem quadrupole mass spectrometer (TQD) with an electrospray ionization (ESI) source. Separation of polyphenols was performed using a 1.7 µm, 100 mm × 2.1 mm UPLC BEH RP C18 column (Waters, Milford, MA, USA). For separation, the mobile phase consisted of 0.1% formic acid in water, *v*/*v* (solvent A), and 0.1% formic acid in 40% acetonitrile, *v*/*v* (solvent B). The flow rate was kept constant at 0.35 mL/min for a total run time of 8 min. The system was run with the following gradient program: from 0 min 5% B, from 0 to 8 min linear to 100% B, and from 8 to 9.5 min for washing and back to initial conditions. The injection volume of the samples was 5 µL, and the column was supported at 50 °C. The following TQD parameters were used: cone voltage of 30 V, capillary voltage of 3500 V, source and desolvation temperature of 120 °C and 350 °C, respectively, and desolvation gas flow rate of 800 L/h. Characterization of the individual polyphenolic compounds was performed on the basis of the retention time, mass-to-charge ratio, fragment ions, and comparison with data obtained with commercial standards and literature findings. The obtained data were processed in Waters MassLynx v.4.1 software (Waters, Milford, MA, USA). The method was validated for parameters such as linearity, accuracy (relative error, RE), limit of detection (LOD), limit of quantification (LOQ), and precision (relative standard deviation, RSD). Quantification was determined by the injection of solutions of known concentrations ranging from 0.05 to 5 mg mL−1 (R 2 ≤ 0.999) of the following phenolic compounds as standards: caffeic acid, caftaric acid, p-coumaric acid, ferulic acid, rosmarinic acid, apigenin 8-*C*-glucoside, (vitexin), kaempferol 3-*O*-glucoside (Extrasynthese, Genay Cedex, France). Stock standard solutions of polyphenols were prepared using methanol. Six calibrators established the peak area ratio of each polyphenol versus the nominal concentration. The regression equation was obtained by a weighted (1/c2) least-squares linear regression. The LOD was determined as a signal-to-noise ratio (S/N) of 3:1, and the LOQ was determined as an S/N of >10. An acceptable RE within ±20% and the intra- and inter-day variations were determined using relative standard deviation (RSD) values, which were determined using relative standard deviation (RSD) values, which were <3.5% for all the analyzed compounds.

#### 4.2.2. Analysis of Volatile Components

The qualitative and quantitative analysis of volatile substances was performed using the HS-SPME solid-phase microextraction method of compound isolation using 100 µm polydimethylsiloxane (PDMS) fiber from Supelco Ltd. (Bellefonte, PA, USA). Next, the analytes were separated and identified using the GC-MS method with the protocol described previously [47]. Briefly, the analyzed material was placed in a 100 mL conical flask equipped with an aluminum membrane. The fiber exposure was carried out using the headspace method for 30 min at 20 °C. Then, the SPME holder was transferred to the gas chromatograph injector (temp. 250 °C). Using a gas chromatograph (GC-MS, Varian 450GC coupled with 240 MS detector, the composition of compounds desorbed from the SPME fiber was examined. Separation of the analytes was carried out using a 30 m × 0.25 mm × 0.25 µm capillary column with a moderately polar HP-5 stationary phase. The column oven temperature program was as follows: start 50 °C for 5 min isotherm, then set to a temperature gradient of 10 °C/min to 300 °C (5 min isotherm). Based on NIST.08 and the Willey database, compounds found in the extracts were identified. GC-MS analysis was performed in duplicate. 

### 4.3. Antioxidant Activity and Total Phenolic Content

The antioxidant activity of the extracts was assessed in the test: (a) ferric ion reduction (FRAP), (b) scavenging of ABTS^•+^ and DPPH^•^ radicals. The reduction in ferric ions in the FRAP test was determined by the method described by Benzie and Strain [48]. An amount of 3 mL of FRAP solution was added to 0.5 mL of the sample. After 10 min of reaction, the absorbance was measured at a wavelength of 593 nm. Absorbance measurement was performed using a UV-VIS spectrometer (Type UV2900, Hitachi, Tokyo, Japan). The scavenging activity of the extracts against ABTS^•+^ radicals was determined by the method described by Re et al. [49]. An amount of 3 mL ABTS^•+^ solution (diluted to an absorbance of 0.7) was added to 0.03 mL of the lavender leaf and flower extracts. After 6 min of incubation, the absorbance at 734 nm was measured using a spectrophotometer. The results are expressed as µmol Trolox equivalent (TE)/g. The ability of the extracts to scavenge DPPH˙ radicals was performed based on the method presented by Blois [50]. To the extracts, 2 mL of methanol DPPH solution was mixed. After 10 min of incubation, the absorbance was measured at 517 nm. Results are expressed as µmol Trolox equivalent (TE)/g.

The total phenolic content (TPC) was determined using the method described by Gao et al. [51]. Measurements of 2 mL distilled water, 0.2 mL Folin–Ciocalteau reagent, and 1 mL 20% sodium carbonate solution were added to the 0.1 mL lavender leaf and flower extracts. After 60 min of incubation, the absorbance was measured at 765 nm. Results are expressed in mg gallic acid (GAE)/g. The results of all analyses were performed in triplicate.

### 4.4. Assessment of the Growth of Mold Fungi

The test was performed on the fungi *Trichoderma viride* Pers., strain A-102, and *Chaetomium globosum* Kunze, strain A-141 (ATCC 6205). Extracts in amounts of 0.5, 1.0, 2.5, and 5 cm^3^ were added to a sterile Petri dish and then poured with an appropriate portion of the microbiological medium so that the total volume of the mixture was 10 cm^3^. An amount of 2.5% maltose–agar medium was used (Bio-Maxima, Lublin, Poland). After 24 h, 5 mm of fungal inoculum was placed in the central part of the Petri dish. The culture was performed in a Thermolyne Type 42000 thermal incubator (ThermoFisher Scientific, Waltham, MA, USA) under temperature and relative humidity conditions of 26 ± 2 °C and 65 ± 2%, respectively. At 48 h intervals, measurements of the growth diameter of the fungus were made in two perpendicular directions. The tests were completed on the day when the Petri dish was completely covered with control samples. In control samples, 5.0 cm^3^ of 60% ethanol was used instead of the extract. Each test was performed in triplicate.

### 4.5. Assessment of the Viability of Microorganisms

The following strains of microorganisms were used for research: *P. aeruginosa* (ATCC 27853), *S. aureus* (ATCC 25923), and *C. albicans* (ATCC 10231). The microorganisms were cultured in Mueller–Hinton broth (Bio-Maxima, Lublin, Poland) and incubated in a shaking incubator at 37 °C overnight. Prior to experiments, microbial cells were brought to a dedicated concentration by dilution in sterile distilled saline based on the McFarland scale. Lavender ethanol extracts at a concentration of 5% were diluted in a 24-well plate in culture medium to concentrations of 2.5, 1.25, 0.625, 0.3125, and 0.156%. Then, 10 µL of the suspension of the tested microorganisms (OD = 0.5 on the McFarland scale) was added to each well. After 24 h of incubation at 37 °C in a rotary incubator, cells from each well were collected and centrifuged at 2000 rpm for 10 min. The supernatant was discarded, and the microorganisms were suspended in 1 mL of PBS and pipetted into a 96-well plate. Two viability tests were performed on the microorganism suspensions prepared in this way: XTT (Cell Proliferation Kit II, Merck, Darmstadt, Germany) and Presto Blue (PrestoBlue™ Cell Viability Reagent, Invitrogen, Waltham, MA, USA). The tests were carried out in accordance with the manufacturer’s recommendations. Each test was performed in triplicate.

### 4.6. Statistical Analysis

Statistical analysis of the results was carried out in Statistica version 13 (TIBCO Software Inc., Palo Alto, CA, USA). Analysis of variance (ANOVA) was used to test (ɑ = 0.05) for significant differences between factors. A comparison of the means was performed by Tukey test, with ɑ = 0.05. In order to multidimensional imaging of data, heat maps were created with the use of R Studio program.

## 5. Conclusions

Based on the obtained research results, that the following conclusions should be made:

1. *L. angustifolia* ecotypes grown in southern Poland are characterized by good biological activity, expressed in terms of effects on microbial growth and viability and antioxidant activity.
2. The leaves and flowers of harvested lavender are a good raw material for many industries, including cosmetics and pharmaceuticals.
3. The obtained ecotypes can be a good alternative to *L. angustifolia* cultivars grown in regions with Mediterranean climates.
4. The dominant phytochemical components differ from those that are standardly determined in lavenders of French origin.

It should also be noted that the presented research is an introduction to the characterization of the raw material. In the experiments planned for the future, it would be necessary to assess the influence of climatic and soil conditions on the variability of the analyzed biological characteristics, as well as their verification, allowing the selection of the most effective methods of isolating bioactive substances from the harvested raw material. The possibility of growing lavender in climatic conditions other than those in the Mediterranean region, as well as lavender having good antioxidant and antimicrobial properties, is an important signal for plant breeders. Lavender is one of the raw plant materials highly desired by the cosmetics, perfume, and pharmaceutical industries. Therefore, new ecotypes of lavender harvested in southern Poland can be a good alternative to raw materials from Mediterranean regions.

## Figures and Tables

**Table 1:** Content of phenolic compounds in extracts of leaves and flowers of *Lavandula angustifolia* Mill. ecotypes.

No	Compound	RT [min]	PL1	KC1	PL2	KC2	Type
			[µg/g]				
1	Syringic acid glucoside	1.933	<LOQ	<LOQ	58.86	89.89	phenolic acid
2	Caftaric acid	2.303	727.16	237.05	55.81	74.58	phenolic acid
3	Ferulic acid glucoside I	3.081	5503.81	2349.46	<LOQ	<LOQ	phenolic acid
4	Coumaric acid glucoside I	3.121	<LOQ	<LOQ	884.23	929.20	phenolic acid
5	Caffeic acid	3.255	<LOQ	<LOQ	171.42	167.52	phenolic acid
6	Ferulic acid glucoside II	3.596	6561.19	3981.95	789.30	885.06	phenolic acid
7	Isorhamnetin 3-*O*-rutinoside	3.66	621.76	243.81	<LOQ	<LOQ	flavonoid
8	Apigenin 4′-*O*-glucoside-7-*O*-glucuronide	3.829	<LOQ	<LOQ	305.64	508.76	flavonoid
9	Coumaric acid glucoside II	4.083	657.85	168.57	253.63	425.43	phenolic acid
10	Chicoric acid	4.397	<LOQ	<LOQ	136.89	182.80	phenolic acid
11	Ferulic acid glucoside III	4.563	2774.17	1303.84	810.97	980.55	phenolic acid
12	Isorhamnetin 3-*O*-rhamnoside	4.775	1102.09	447.61	215.45	136.42	flavonoid
13	(+)Catechin-rhamnoside-pentoside	4.888	<LOQ	<LOQ	194.95	174.18	flavonoid
14	Salvinic acid B	5.305	<LOQ	<LOQ	75.54	206.99	phenolic acid
15	Apigenin *C*-glucoside	5.436	<LOQ	<LOQ	199.70	140.83	flavonoid
16	Rosmarinic acid	5.623	1766.99	348.29	1229.33	829.68	phenolic acid
17	Ferulic acid	5.950	402.20	133.13	11.15	<LOQ	phenolic acid
18	Unidentified caffeic acid derivative	6.064	<LOQ	<LOQ	16.35	24.30	phenolic acid
19	Kaempferol	6.696	<LOQ	<LOQ	17.59	17.34	flavonoid
20	Undefined caffeic acid derivative	7.036	349.66	257.43	26.54	15.56	phenolic acid
21	Undefined caffeic acid derivative	7.333	<LOQ	<LOQ	57.99	39.97	phenolic acid
22	Apigenin	7.601	<LOQ	<LOQ	11.70	16.82	flavonoid

**Table 2:** Results of GC-MS analysis—qualitative and quantitative differences in the composition of volatile substances between *Lavandula angustifolia* Mill. leaf extracts (PL1 and KC1) and flower extracts (PL2 and KC2).

No.	RT [min]	Peak Share in the Chromatogram [%]	Ordinary Substance Name	RT [min]	Peak Share in the Chromatogram [%]	Ordinary Substance Name
	PL1			KC1		
1	8.72	3.86	β-Pinene	7.22	4.40	α-Pinene
2	9.45	15.87	3-Carene	8.08	8.30	Camphene
3	9.69	10.16	p-Cymene (cymene isomers mix)	8.73	6.63	β-Pinene
4	9.74	22.67	m-Cymene (cymene isomers mix)	9.23	2.93	Decane
5	983	23.82	Limonene	9.45	16.15	3-Carene
6	9.89	10.62	Eucalyptol	9.69	9.42	p-Cymene (cymene isomers mix)
7	10.00	5.30	Ocimene isomers mix	9.74	18.79	m-Cymene (cymene isomers mix)
8	17.25	7.66	γ-cadinene	9.83	24.81	Limonene
9	-	-	-	9.89	8.53	Eucalyptol
	PL2			KC2		
1	7.22	1.38	α-Pinene	8.58	1.23	3-octanone
2	8.08	1.01	Camphene	8.70	3.74	β-Pinene
3	8.72	1.63	β-Pinene	9.47	1.72	o-Cymene
4	9.05	4.31	Myrcene	9.57	2.49	(±)-Limonene
5	9.45	1.71	3-Carene	9.63	1.54	Eucalyptol
6	9.74	2.02	m-Cymene (cymene isomers mix)	9.78	8.98	Ocimene isomers mix
7	9.83	6.09	Limonene	10.01	6.40	Ocimene isomers mix
8	10.01	41.33	Ocimene isomers mix	10.21	1.75	Ocimene isomers mix
9	10.21	8.61	Ocimene isomers mix	11.03	3.83	Linalool
10	11.16	1.60	Linalool	11.25	3.12	1-Octen-3-yl acetate
11	11.36	1.50	1-Octen-3-yl acetate	13.63	42.62	Linalyl acetate
12	11.67	1.51	2,6-Dimethyl-2,4,6-octatriene	15.43	1.99	Lavandulyl acetate
13	13.67	17.55	Linalyl acetate	16.02	3.15	α-santalene
14	16.03	2.42	α-santalene	16.08	13.41	β-Caryophyllene
15	16.09	7.12	β-Caryophyllene	16.41	1.13	β-Farnesene
16	-	-	-	17.25	2.81	γ-cadinene

**Table 3:** Total phenolic content and antioxidant activity of lavender flowers (PL2 and KC2) and leaves (PL1 and KC1).

No.	Sample	Total Phenolic Content (TPC)	ABTS^•+^ Radical Scavenging Activity	DPPH^•^ Radical Scavenging Activity	Ferric Reducing Antioxidant Power Assay (FRAP)
		mg GAE/g	μmol (TE)/g		
**1**	PL1	24.49 ± 0.45 ^c^	177.75 ± 0.49 ^d^	164.88 ± 2.34 ^c^	89.10 ± 0.45 ^c^
**2**	KC1	9.70 ± 0.712 ^a^	136.52 ± 0.70 ^c^	63.34 ± 1.42 ^a^	31.47 ± 1.32 ^a^
**3**	PL2	15.84 ± 0.56 ^b^	125.05 ± 2.47 ^b^	82.35 ± 0.96 ^b^	54.64 ± 0.51 ^b^
**4**	KC2	16.95 ± 0.48 ^b^	82.27 ± 2.47 ^a^	86.70 ± 2.25 ^b^	55.49 ± 0.13 ^b^

^a,b,c,d^ are homogeneous groups by the Tukey test.

**Table 4:** Analysis of variance in terms of the significance of the impact of selected factors: ecotype (PL and KC), type of extract (F/L-flowers/leaves), and the interactions between them.

	*p*-Value	X
Total phenolic content		
ecotype	0.038 × 10^−7^	42.1
extract L/F	0.025	0.4
ecotype × extract L/F	0.011 × 10^−7^	57.0
Error		0.5
ABTS^•+^		
ecotype	0.019 × 10^−7^	38.0
extract L/F	0.028 × 10^−8^	61.6
ecotype × extract L/F	0.601	0.1
Error		0.3
DPPH^•^		
ecotype	0.056 × 10^−9^	39.0
extract L/F	0.029 × 10^−7^	14.5
ecotype × extract L/F	0.028 × 10^−9^	46.3
Error		0.2
FRAP		
ecotype	0.032 × 10^−10^	47.7
extract L/F	0.020 × 10^−4^	1.6
ecotype × extract L/F	0.025 × 10^−10^	50.6
Error		0.1

*p*—probability of error, X—percentage influence of factors (ecotype, type of extract L/F, or/and the interactions between them).

**Table 5:** Growth of *T. viride* Pers. on a medium containing various amounts of lavender extracts.

Plant Material	Concentration of Extracts in Growth Medium (mL/100 mL)	Day of Observation		*p*-Value	α
		2	3		
		Diameter of Mycelium (mm)		Tukey’s Test	
PL 1	statistics F			1.24 × 10^−10^	0.05
	0 (control)	58.8	90.0	a	
	0.5	24.8	30.0	b	
	1.0	0	0	c	
	2.5	0	0	c	
	5.0	0	0	c	
KC 1	statistics F			6.93 × 10^−11^	0.05
	0 (control)	58.8	90.0	a	
	0.5	27.5	33.3	b	
	1.0	0	0	c	
	2.5	0	0	c	
	5.0	0	0	c	
PL 2	statistics F			1.11 × 10^−13^	0.05
	0 (control)	58.8	90.0	a	
	0.5	26.7	29.2	b	
	1.0	11.0	11.8	c	
	2.5	0	0	d	
	5.0	0	0	d	
KC 2	statistics F			4.22 × 10^−13^	0.05
	0 (control)	58.8	90.0	a	
	0.5	47.2	66.2	b	
	1.0	13.3	14.2	c	
	2.5	0	0	d	
	5.0	0	0	d	

“a, b, c, d” are homogeneous groups by the Tukey test; *p*-value—probability of error; α—statistical significance level.

**Table 6:** Growth of *C. globosum* Kunze on a medium containing various amounts of lavender extracts.

Plant Material	Concentration of Extracts in Growth Medium (mL/100 mL)	Day of Observation					*p*-Value	α
		2	3	5	7	9		
		Diameter of Mycelium (mm)					Tukey’s Test	
PL1	statistics F						8.17 × 10^−8^	0.05
	0 (control)	16.0	20.3	28.8	40.2	44.8	a	
	0.5	0.7	1.5	2.0	2.0	2.0	b	
	1.0	0	0	0	0	0	b	
	2.5	0	0	0	0	0	b	
	5.0	0	0	0	0	0	b	
KC1	statistics F						4.56 × 10^−8^	0.05
	0 (control)	16.0	20.3	28.8	40.2	44.8	a	
	0.5	1.0	1.5	1.7	1.7	1.7	b	
	1.0	0	0	0	0	0	b	
	2.5	0	0	0	0	0	b	
	5.0	0	0	0	0	0	b	
PL2	statistics F						5.52 × 10^−8^	0.05
	0 (control)	16.0	20.3	28.8	40.2	44.8	a	
	0.5	0.3	0.3	1.5	1.5	1,5	b	
	1.0	0	0	0	0	0	b	
	2.5	0	0	0	0	0	b	
	5.0	0	0	0	0	0	b	
KC2	statistics F						3.59 × 10^−8^	0.05
	0 (control)	16.0	20.3	28.8	40.2	44.8	a	
	0.5	0.3	0.3	0.3	0.3	0.3	b	
	1.0	0	0	0	0	0	b	
	2.5	0	0	0	0	0	b	
	5.0	0	0	0	0	0	b	

“a, b” are homogeneous groups by the Tukey test; *p*-value—probability of error; α—statistical significance level.

## Data Availability

No new data were created or analyzed in this study. Data sharing is not applicable to this article.
